# Autonomic Modulation in Duchenne Muscular Dystrophy During a Computer Task: A Prospective Transversal Controlled Trial Assessment by Non-linear Techniques

**DOI:** 10.3389/fneur.2021.720282

**Published:** 2021-11-23

**Authors:** Mayra Priscila Boscolo Alvarez, Carlos Bandeira de Mello Monteiro, Talita Dias da Silva, Vitor E. Valenti, Celso Ferreira-Filho, Annette Sterr, Luiz Carlos Marques Vanderlei, Celso Ferreira, David M. Garner

**Affiliations:** ^1^Programa de Pós-Graduação em Ciências da Reabilitação, Faculdade de Medicina da Universidade de São Paulo (FMUSP), São Paulo, Brazil; ^2^Escola de Artes, Ciências e Humanidades da Universidade de São Paulo (EACH/USP), São Paulo, Brazil; ^3^Departamento de Medicina (Cardiologia), Escola Paulista de Medicina, Universidade Federal de São Paulo (EPM/UNIFESP), São Paulo, Brazil; ^4^Faculdade de Medicina, Universidade Cidade de São Paulo (UNICID), São Paulo, Brazil; ^5^Department of Studies on the Autonomic Nervous System, Faculty of Science and Technology, Universidade Estadual Paulista “Júlio de Mesquita Filho” (UNESP), São Paulo, Brazil; ^6^School of Psychology, University of Surrey, Guildford, United Kingdom; ^7^Cardiorespiratory Research Group, Department of Biological and Medical Sciences, Faculty of Health and Life Sciences, Oxford Brookes University, Oxford, United Kingdom

**Keywords:** heart rate variability, Duchenne Muscular Dystrophy, autonomic nervous system, Chaotic Global Techniques, health care technology

## Abstract

**Introduction:** Due to functional and autonomic difficulties faced by individuals with Duchenne Muscular Dystrophy (DMD), the use of assistive technology is critical to provide or facilitate functional abilities. The key objective was to investigate acute cardiac autonomic responses, by application of Heart Rate Variability (HRV), during computer tasks in subjects with DMD via techniques based on non-linear dynamics.

**Method:** HRV was attained via a Polar RS800CX. Then, was evaluated by Chaotic Global Techniques (CGT). Forty-five male subjects were included in the DMD group and age-matched with 45 in the healthy Typical Development (TD) control group. They were assessed for 20 min at rest sitting, and then 5 min whilst performing the maze task on a computer.

**Results:** Both TD and DMD subjects exhibited a significantly reduced HRV measured by chaotic global combinations when undertaking the computer maze paradigm tests. DMD subjects presented decreased HRV during rest and computer task than TD subjects.

**Conclusion:** While there is an impaired HRV in subjects with DMD, there remains an adaptation of the ANS during the computer tasks. The identification of autonomic impairment is critical, considering that the computer tasks in the DMD community may elevate their level of social inclusion, participation and independence.

## Introduction

Duchenne Muscular Dystrophy (DMD), is the most prevalent form of muscular dystrophy (1). It is a terminal illness characterized by progressive muscular weakness with proximal onset in the lower limbs spreading later to the rest of the body, which leads the individual to become confined to a wheelchair (2). In addition, cardiorespiratory (3), and autonomic (4) changes are observed and death in early adulthood, at around 20 years old (2).

In consideration of the progressive functional difficulties presented by people with DMD, the use of assistive technology by rehabilitation programs is important as it promotes greater functional independence and improves the social performance of these disabled persons (5). With computational advances in assistive technology, rehabilitation programs via computing situations during treatment allow the persons with DMD to practice tasks in a different environment using an informal interface and rapid responses. It is similarly capable of providing a dynamic interaction and vivacity with elements and goals, by means of logical reasoning and reaction times associated with the movement, permitting the repetition of muscle contractions and improved performance (6). In this manner, studying the acute and chronic responses of altered physiological systems in DMD individuals despite this technology is fundamental and could support a better understanding of these changes induced by DMD and, an improved guide as to it becoming a therapeutic procedure.

As described previously, an important physiological system modified by DMD is the autonomic nervous system (ANS) (7). Studies including the acute cardiac autonomic responses during computer tasks in DMD are rare. Yet, by implying the linear methods for analysis of heart rate variability (HRV) to evaluate cardiac autonomic responses in DMD subjects during computer tasks, Alvarez et al. (8) detected that DMD subjects responded with a reduced HRV. But then, those during computer tasks exhibited greater intensity of cardiac autonomic responses when compared to the Typical Developed (TD) subjects which were the control group.

Nevertheless, Sassi et al. (9) stated that non-linear techniques of HRV may provide new evidence for the understanding of cardiac control, as it provides information on the complexity of the underlying physiological mechanisms. An innovative group of procedures sensitive non-linearly for the detection of HRV fluctuations that allows us to understand the irregularity, unpredictability, extent of fractal self-similarity, and complexity of the signals are the Chaotic Global Techniques (CGT) (10). These metrics have been effectively applied to assess cardiac autonomic modulation in physiological and pathological conditions (11–15), once non-linear methods describe complex rhythm fluctuations more appropriately than linear methods (16), as “chaos theory” exhibits characteristics that are consistent with those found physiologically in the human organism.

The human organism exhibits chaotic properties, specifically, it is composed of several systems, which are dynamic, deterministic, and sensitive to initial circumstances. All these factors are capable of producing non-linear, non-proportional, or direct responses to stimuli in these systems, hence, trivial dysfunctions in one organ can engender different levels of dysfunction in the others (17, 18). Thus, the human body is better understood as a complex and non-linear system, hence assessments of HRV via non-linear techniques have gained recognition (19).

Accordingly, we proposed to study acute cardiac autonomic responses during computer tasks in individuals with DMD vs. TD people using CGT. The CGT may complement important information in the research literature on the complexity of the cardiac autonomic response during the execution of computer games. We hypothesized that during computer tasks the HRV responses *via* CGT demonstrated a lesser response to complexity. Subsequently, it should have a greater response in the DMD subjects.

## Materials and Methods

This trial was completed using the data analyzed and published by Alvarez et al. (8), yet, for this study the data were evaluated through CGT. The present study was performed in accordance to the Declaration of Helsinki.

This study conforms to the Consolidated Standards of Reporting Trials (CONSORT) statement (20).

### Ethical Approval and Informed Consent

The study protocol was approved by the research ethics committee of the University of São Paulo, reference number 236/13 was attached. Written consent terms were obtained from participants (or guardians) over 18 years old. Acceptance terms were obtained from participants younger than 17 and also written consent terms from the legally authorized representative/parents of those participants under 17 years old.

### Participants

Forty-five individuals with DMD and 45 age-matched healthy TD individuals participated in the trial. DMD diagnoses was based on molecular methods and/or muscular protein expression. Exclusion criteria comprised subjects with severely dilated myocardium, other associated diseases and individuals with inability to understand task instructions. DMD severity was classified according to the Vignos scale (21). See Table 1.

**Table 1:** Disease status according to Vignos scale.

**Vignos scale**	**Description of DMD patients mobility at the specified Vignos scale level**.	**No of patients**
	**The Vignos scales from zero to ten (0–10)**	
1	Walks and climbs stairs without assistance	4
2	Walks and climbs stairs with aid of railing	4
3	Walks and climbs stairs slowly with aid of railing (more than 25 s for eight standard steps)	1
4	Walks unassisted and rises from chair; cannot climb stairs	1
5	Walks unassisted; cannot rise from chair; cannot climb stairs	0
6	Walks only with assistance or walks independently with leg braces	0
7	Walks in leg braces but requires assistance for balance	19
8	Maintains standing with leg braces but is unable to walk even with assistance	14
9	In wheelchair	2
10	Confined to bed	0

### Data Collection

The protocol was identical to that used in Alvarez et al. (8) where HR was recorded beat-to-beat (RR intervals) using the portable Polar RS800CX eletrocardiographic monitor (Polar Electro, Finland). HR was recorded prior to the onset and at the end of the 5 min of the computer maze paradigm task.

**Figure 1:**
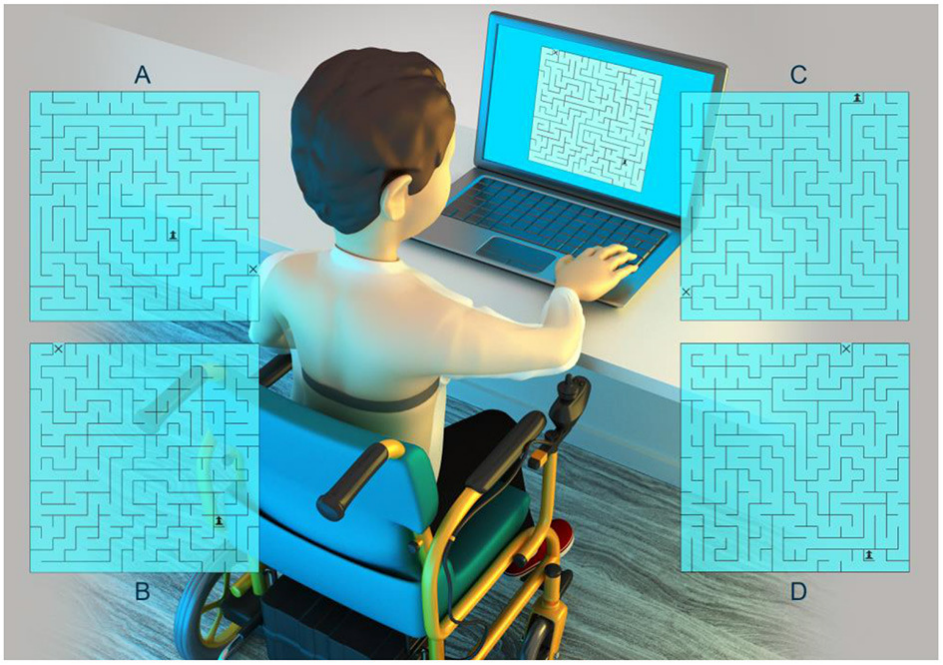
Visualization of experimental set up. Mazes **(A–D)** represent the four mazes used in this study. The range was of one to four mazes performed for the participants during 5 minutes.

The subjects were seated in a standard chair (walkers, TD group, and DMD) or in their own wheelchair (non-walkers, DMD group), the Polar watch was situated on their wrist. The analysis of HRV was possible via the recording of RR interval in two periods: the period of 20 min at rest seated, and then during the computer task for 5 min, as demonstrated in Figure 1.

The computer task used a maze paradigm with one correct pathway that could be negotiated and ultimately solved; the paradigm was adapted by Souza et al. (22). All participants were positioned comfortably with an evaluator responsible for instruction and data annotation.

Each individual was instructed to walk the correct path with the digital character pawn (pointed on the screen by the evaluator) to the exit of the maze identified by an “x” (pointed on the screen by the evaluator). It was provided for the subjects, who used the arrow buttons on the keyboard, identified by up, down, right and left, using the dominant hand, with the arrows moving through a 20 cm × 20 cm maze. Participants were told to complete the maze as quickly as possible.

### HRV Analysis

HRV analysis followed the guidelines published by the Task Force of the European Society of Cardiology and North American Society of Pacing and Electrophysiology (23). The RR intervals were recorded, and then were downloaded to the Polar Precision Performance program (v.3.0). This software enabled the visualization of HR and the extraction of a cardiac period (RR intervals series; the variation of beat-to-beat interval in milliseconds) file in “.txt” format. For analysis of HRV data at rest, we evaluated 1,000 consecutive RR intervals, and for HRV analysis for the computational task, the number of consecutive RR intervals obtained was exactly 256 RR intervals. Digital filtering complemented by manual filtering was performed to eliminate artifacts and only series with >95% of sinus beats were included in the study (19). HRV analysis was undertaken through CGT.

### CGT Analysis

As stated in 2016 by Wajnsztejn et al. (12) and, later by Alves et al. (24), these techniques encompass of a variety of mathematical and signal processing events. In this study, we enforced the Multi-Taper Method (MTM) power spectral technique (12, 24) to generate *high spectral* Entropy (*hs*Entropy) (24), *high spectral* Detrended Fluctuation Analysis (*hs*DFA) (25), and the Spectral Multi-Taper Method (sMTM) (10). From these three chaotic global values we computed seven non-trivial combinations which we termed the Chaotic Forward Parameters (CFP1 to CFP7) (26–29). For further information regarding the MTM techniques it is suggested to refer to Percival and Walden (30) or Thomson (31).

### Normality and Levene's Test

The dependent variables (CFP1 to CFP7), and the independent variables “group” (DMD and TD) and “task,” at (rest or computer task through maze paradigm). Parametric statistics assume the datasets are normally distributed, so the use of the mean as a measure of central tendancy. Consequently, the first assumption for the two-way analysis of variance test is normality. We applied the Anderson-Darling (26) and the Lilliefors (27) tests. The Anderson-Darling test for normality applies an empirical cumulative distribution function. The Lilliefors test is useful in studies with small sample sizes (*n* < 20). The results of the tests for normality were borderline. Nonetheless, as there is no non-parametric alternative to the two-way analysis of variance test we proceed aware that the accuracy could be flawed. For this reason we raise the level of significances to *p* < 0.01 (or <1%), rather than the usual *p* < 0.05 (or <5%). The second assumption was that of the equality of variances by Levene's test (32, 33). Hence, the dependent variables were submitted to a 2 (group: DMD, TD) by 2 (Task: Rest, Computer) Multiple Analysis of Variance (MANOVA) with Repeated Measures (RM) on the last factor for each CFP index. *Post-hoc* comparisons were undertaken by Tukey-LSD (Least Significant Difference) test. Partial eta-squared ($\eta_{\text{p}}^{2}$) was reported to measure the effect sizes and were interpreted as small (effect size >0.01), medium (effect size >0.06), or large (effect size >0.14) (34, 35); additionally we reported the observed power (OP). The software package operated was SPSS, version 26.0 (Chicago, Illinois, USA).

## Results

### Anthropometric Data and Medications

Table 2 presents the anthropometric data and Table 3 lists the medications taken by the DMD group. The TD group did not take any medications.

**Table 2:** Values (mean ± standard deviation) of age, height, mass, and body mass index (BMI) for both TD and DMD groups.

**Variable**	**TD-group**	**DMD-group**	***p-*value**
Age (years)	15.4 ± 2.8	15.4 ± 2.9	0.455
Height (m)	1.68 ± 0.12	1.56 ± 0.17	<0.001
Mass (kg)	63.2 ± 15.5	55.84 ± 17.9	0.013
BMI (kg/m^2^)	20.04 ± 3.72	22.42 ± 4.71	0.331

*TD, Typical Development; DMD, Duchenne Muscular Dystrophy; BMI, body mass index; m, meters; kg, kilograms; kg/m^2^, kilograms per square meter*.

**Table 3:** The cardiac medications for DMD-group.

**Medication on DMD-group**	**Number of patients (%)**
Beta-blockers	13 (28.89)
ACE-inhibitor	5 (11.11)
Beta-blockers + ACE-inhibitors	20 (44.44)
Corticosteroids	44 (97.77)
No medication	7 (15.56)

*ACE-inhibitors, angiotensin-converting enzyme inhibitors*.

### Normality and Levene's Test

The results of the Levene's test of equality, are illustrated in Table 4. A significant *p*-value is set at > 0.05 (*p* > 0.05, >5%). This ensures that similar variances can be assumed or else the ANOVA test is invalid. This was achieved by the all of the chaotic global combinations applied with the exception of CFP7.

**Table 4:** Levene's test of equality of error variances: *F*-value and significances of the seven permutations of chaotic global parameters (CFP1 to CFP7).

**CFPx**	***F***	**Sig**.
CFP1	1.221	0.304
CFP2	0.806	0.492
CFP3	0.410	0.746
CFP4	0.606	0.612
CFP5	1.572	0.198
CFP6	0.707	0.549
CFP7	4.159	0.007

*This tests the null hypothesis that the error variances of the dependant variable is equal across groups*.

### Repeated Measures-Multiple Analysis of Variance

Significant effects for Group [Wilks Lambda = 0.006; *F*_(7, 82)_ = 1897.1; *p* < 0.001; $\eta_{\text{p}}^{2}$ = 0.99: OP: 1.00], Task [Wilks Lambda = 0.004; *F*_(7, 82)_ = 2739.2; *p* < 0.001; $\eta_{\text{p}}^{2}$ = 0.99: OP: 1.00] and significant interaction between Group and Task [Wilks Lambda = 0.032; *F*_(7, 82)_ = 352.1; *p* < 0.001; $\eta_{\text{p}}^{2}$ = 0.97: OP: 1.00]. The separate ANOVAs that established differences between Groups and Tasks in each CFP, besides their Mean, Standard Deviation, Confidence Interval, and *post-hoc* values are described in Table 5.

**Table 5:** The table below demonstrates the results of a Repeated Measures-Multiple Analysis of Variance (RM-MANOVA), between Groups, within Tasks and its interactions, in addition to mean, stardard deviation, and confidence intervals for each CFP.

		**Rest**	**Computer task**		**Main effect task** **(*df*: 1, 88)**	**Main effect group** **(*df*: 1, 88)**	**Interaction taks × group** **(*df*: 1, 88)**
	**Groups**	**Mean** **±** **SD [95% CI]**	**Mean** **±** **SD [95% CI]**	***p-*****value**^**§**^ **(Rest** **×** **Computer)**	***F*****;** ***p*****-value;** $\eta_{\text{p}}^{2}$**; O.P**.	***F*****;** ***p*****-value;** $\eta_{\text{p}}^{2}$**; O.P**.	***F*****;** ***p*****-value;** $\eta_{\text{p}}^{2}$**; O.P**.
CFP1	DMD	0.8371 ± 0.1270 [0.798; 0.877]	0.7817 ± 0.1189 [0.745; 0.819]	** <0.001**↓	57.1; *** <0.001;*** 0.39; 1.00	1.13; 0.289; 0.01; 0.18	1.14; 0.288; 0.01; 0.19
	TD	0.8739 ± 0.1399 [0.834; 0.914]	0.8003 ± 0.1318 [0.763; 0.837]	** <0.001**↓			
	*p*-value^§^ (DMD × TD)	0.194	0.484				
CFP2	DMD	0.6116 ± 0.1106 [0.583; 0.640]	0.5942 ± 0.0988 [0.567; 0.621]	** <0.001**↓	1.70; *0.196;* 0.02; 0.25	0.62; 0.434; 0.01; 0.12	15.4; *** <0.001;*** 0.16; 0.97
	TD	0.6003 ± 0.0811 [0.572; 0.629]	0.6349 ± 0.0829 [0.608; 0.662]	0.067			
	*p-*value^§^ (DMD × TD)	0.580	0.037*				
CFP3	DMD	0.7463 ± 0.1128 [0.712; 0.781]	0.6655 ± 0.1131 [0.630; 0.701]	** <0.001**↓	185.8; *** <0.001;*** 0.68; 1.00	15.6; *** <0.001;*** 0.15; 0.97	16.9; *** <0.001;*** 0.16; 0.98
	TD	0.6889 ± 0.1201 [0.654; 0.723]	0.5383 ± 0.1252 [0.503; 0.574]	** <0.001**↓			
	*p-*value^§^ (DMD × TD)	0.022*	** <0.001**				
CFP4	DMD	0.6586 ± 0.2118 [0.600; 0.717]	0.6279 ± 0.1960 [0.572; 0.683]	** <0.001**↓	18.1; *** <0.001;*** 0.17; 0.99	13.9; *** <0.001;*** 0.14; 0.96	2.65; 0.107; 0.03; 0.36
	TD	0.8231 ± 0.1831 [0.764; 0.882]	0.7541 ± 0.1789 [0.669; 0.810]	0.067			
	*p*-value^§^ (DMD × TD)	** <0.001**	**0.002**				
CFP5	DMD	0.3500 ± 0.1589 [0.308; 0.392]	0.3832 ± 0.1524 [0.341; 0.426]	** <0.001**↑	22.3; *** <0.001;*** 0.20; 1.00	42.6; *** <0.001;*** 0.33; 1.00	0.90; 0.345; 0.01; 0.16
	TD	0.5289 ± 0.1216 [0.487; 0.571]	0.5789 ± 0.1331 [0.536; 0.621]	**0.009**↑			
	*p*-value^§^ (DMD × TD)	** <0.001**	** <0.001**				
CFP6	DMD	0.5530 ± 0.1591 [0.508; 0.598]	0.4929 ± 0.1407 [0.452; 0.534]	** <0.001**↓	135.8; *** <0.001;*** 0.61; 1.00	1.10; 0.296; 0.01; 0.18	24.6; *** <0.001;*** 0.22; 1.00
	TD	0.6284 ± 0.1475 [0.583; 0.674]	0.4793 ± 0.1353 [0.438; 520]	** <0.001**↓			
	*p*-value^§^ (DMD × TD)	0.022*	0.641				
CFP7	DMD	0.4413 ± 0.2124 [0.388; 0.495]	0.3932 ± 0.1984 [0.341; 0.446]	**0.009**↓	16.1; *** <0.001;*** 0.15; 0.98	33.9; *** <0.001;*** 0.28; 1.00	0.06; 0.814; 0.00; 0.06
	TD	0.2287 ± 0.1439 [0.175; 0.282]	0.1860 ± 0.1530 [0.134; 0.238]	**0.004**↓			
	*p-*value^§^ (DMD × TD)	** <0.001**	** <0.001**				

The significant p-values (<0.01) are exemplified in bold, and the marginally significant results are followed by an *. DMD, Duchenne Muscular Dystrophy group; TD, Typically Developed group; CFP, Chaotic Forward Parameters; SD, standard deviation; CI, confidence interval; df, degrees of freedom; O.P., observed power. ^§^p-value from post-hoc (Tukey-LSD) comparisons; arrows indicate whether the values represent significant decrease (↓) or increase (↑) of the indices from rest to computer task.

### Mean and Standard Deviation

**Figure 2:**
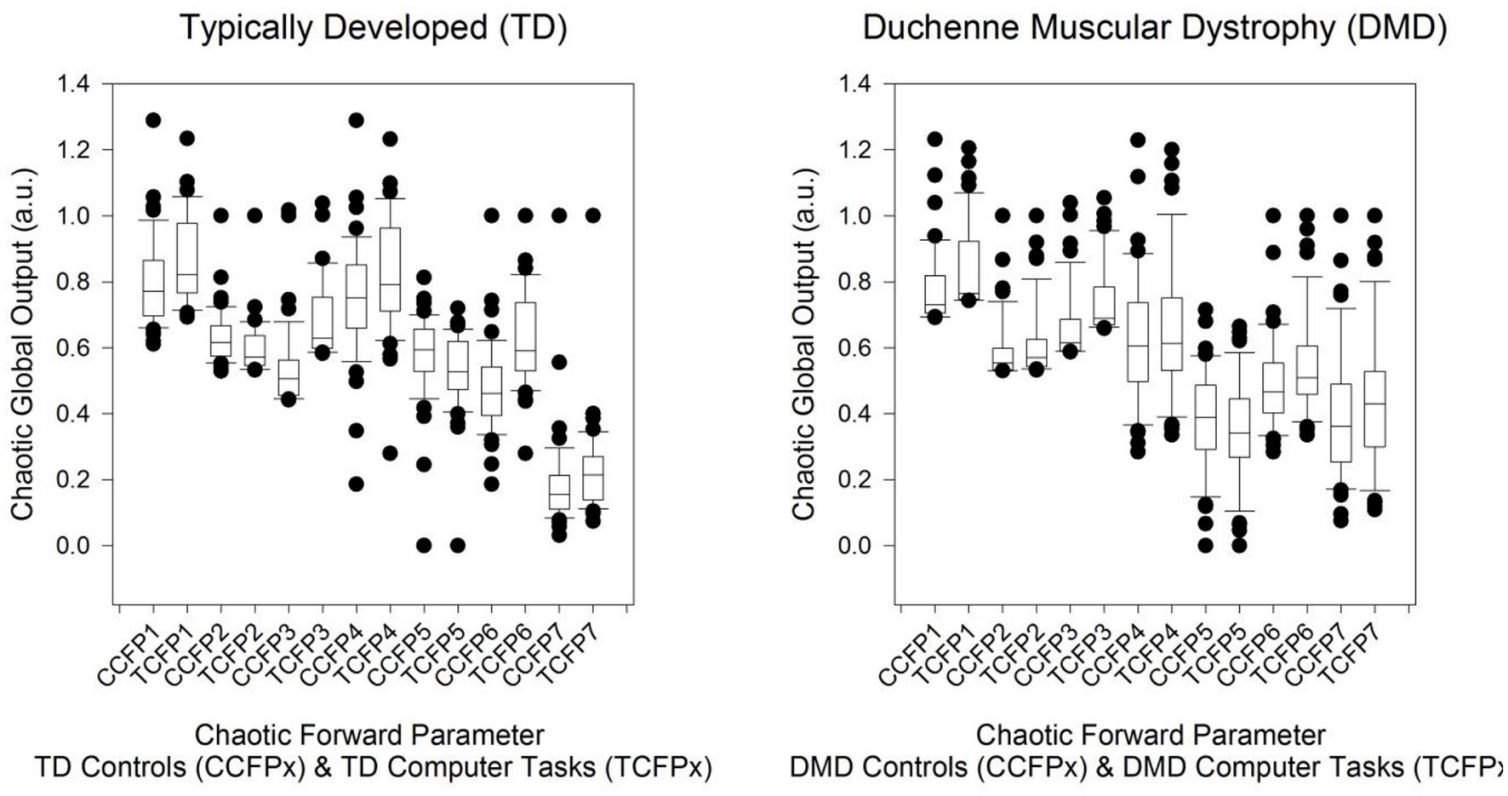
Boxplot (left) for CFP1 to CFP7 of TD subjects RR intervals in TD control subjects' “at rest” and TD control computer task subjects. Boxplot (right) for CFP1 to CFP7 of DMD subjects' RR intervals in DMD control subjects “at rest” and computer task subjects with DMD. The point nearest to the zero is the minimum and the point furthermost away is the maximum. The point next nearest to the zero is the 5th percentile and next furthest away is the 95th percentile. The edge of the box nearest to zero specifies the 25th percentile, and the edge of the box furthest from zero specifies the 75th percentile. The line within the box is the median. Error bars are the 10th and 90th percentiles.

Regarding Figure 2, the boxplot (left) for CFP1 to CFP7 of TD subjects RR intervals' in TD control subjects “at rest” (CCFPx: RR intervals of length 1,000) and TD control computer task subjects (TCFPx: RR intervals of length 256). Next, the boxplot (right) for CFP1 to CFP7 of DMD subjects' RR intervals in DMD control subjects “at rest” (CCFPx: RR intervals of length 1,000) and computer task subjects with DMD (TCFPx: RR intervals of length 256).

The output of the RM-MANOVA can be defined as follows. Initially, deviations in the chaotic global responses in the computer task mode (Table 5). There are significant reductions from rest to computer task CFP1, CFP3, CFP4, CFP6, and CFP7, and an increase for CFP5. Next, we revealed that there are significantly lower responses in DMD group (in both rest and computer task) compared to TD group for CFP3, CFP4, CFP5, and CFP7. Finally, while the interaction term for the computer task and DMD is statistically significant for CFP2, CFP3, and CFP6, the *post-hoc* results are non-significant at a level of significance of *p* <0.01 (or <1%).

### Regression Analysis

To understand which factors may influence the HRV response in DMD subjects, three regression analysis were completed between the dependent variables [(1) use of beta-blockers (yes/no), (2) use of ACE inhibitors (yes/no), and (3) use of corticoids (yes/no)] and independent variables [the differences between values of CFP1 to CFP7 on computer task and at rest, namely Δ of computer task and rest]. The analysis revealed no significant regression models for any of the three dependent variables (see Table 6).

**Table 6:** The table illustrates the regression models and three dependent variables.

**Dependent variables:**	**Beta-blockers**		**ACEi**		**Corticosteroids**	
**Model summary and ANOVA:**	***F***_**(7, 37)**_ **=** **1.96;** ***p*** **=** **0.088;** ***r***^**2**^ **=** **0.27**		***F***_**(7, 37)**_ **=** **0.33;** ***p*** **=** **0.935;** ***r***^**2**^ **=** **0.06**		***F***_**(7, 37)**_ **=** **0.81;** ***p*** **=** **0.582;** ***r***^**2**^ **=** **0.13**	
**Coefficients:**	***ß***	***p*** **-value**	***ß***	***p*** **-value**	***ß***	***p-*** **value**
CFP1	1.44	0.768	−1.789	0.748	−6.644	0.218
CFP2	−4.48	0.127	0.121	0.971	3.539	0.266
CFP3	1.03	0.880	1.959	0.800	8.933	0.234
CFP4	1.26	0.842	−0.152	0.983	6.786	0.331
CFP5	13.48	0.167	5.811	0.596	−6.131	0.560
CFP6	−4.21	0.544	−1.266	0.872	−7.540	0.322
CFP7	13.70	0.104	3.618	0.701	−13.557	0.139

*CFP, Chaotic Forward Parameters; ACEi, angiotensin converting enzyme inhibitors*.

## Discussion

We evaluated HRV responses induced by a computer task in people with DMD and TD. As an important outcome, we established that, while the DMD group offered significantly lower autonomic responses when compared to the TD group in both rest and computer task, DMD and TD groups presented similar patterns of response from rest to computer task; explicitly, intense autonomic responses induced by the task, as established in Table 5. We can postulate that this similar pattern of responses could be due to the condition that it was a task that required more distal muscular effort, with only the need of finger movement (which is mostly maintained in adolescents with DMD), and became more of a cognitive task.

This response of both DMD and TD groups has been previously revealed in the study of Luque-Casado et al. (36), which concluded that cardiac autonomic modulation is highly sensitive to overall demands of sustained attention under the influence of cognitive processes, leading to a decrease of heart rate variability (HRV); then this is the case of the task used in our study that requires sustained attention and cognitive effort (besides the distal muscular effort) to attain the target. This process appears to be the role of the prefrontal cortex in the modulation of subcortical cardio-acceleratory circuits via an inhibitory pathway that is associated with vagal function and that can be indexed by HRV (*via* the baroreceptor system, namely, the negative feedback loop adjusting heart activity to the blood pressure fluctuations) (37, 38).

Considering the general lower autonomic response from DMD here, in this study; in a previous study, Silva et al. (4) through a systematic review about the HRV of subjects with DMD, confirmed that these subjects presented an impaired autonomic modulation with a decreased parasympathetic modulation and, occasionally, increased sympathetic control. Vanderlei et al. (39) revealed in obese young individuals an increase in the chaotic global response, but this is unusual since typically the pathological or disease state reduces HRV through a decreased chaotic global response.

Regarding autonomic responses during the computer task in subjects with DMD, Alvarez et al. (8) suggested the computational tasks can support the functional capacities through training and competence of the ANS, and that cardiac autonomic modulation data is useful for clinical practice. Thus, computer tasks must be performed under supervision and care taken to avoid psychological overload and exacerbation to the ANS, as previous studies have achieved reduced HRV from an early stage of disease in DMD (4, 32, 33), conceivably leading to cardiac (40, 41), or respiratory (42) failure.

Even so, Alvarez et al. (8) evaluated only linear approaches and previous studies have demonstrated that non-linear methods are clinically important for the interpretation of pathological mechanisms related to HRV, providing extra information to using linear methods alone (43). de Godoy (43) concluded, via a review study on patients with cardiovascular disease, that the non-linear analysis of HRV is valuable to characterize autonomic balance, which is a reliable marker of complications and subsequent mortality.

The HRV analysis techniques with linear methods, in the time and frequency domains are not sufficient to characterize a cardiac dynamical balance. Instead, the mechanisms involved in cardiovascular regulation interact with each other in complex and chaotic ways (43). Therefore, HRV analysis using CGT should be able to characterize the HRV in a more reliable manner and, afterwards, autonomic cardiac modulation.

In this study, we have revealed that the computer task reduces the HRV as measured by CGT in the CFP1, CFP3, CFP4, CFP5, CFP6, and CFP7 combinations. This is the case for TD and the DMD groups. It was expected that CFP1 and CFP3 would be two of the most significant parameters. CFP1 applies all three chaotic global parameters. There is evidence to enforce CFP1 as the most robust function as with the optimization study by Garner and Ling (10). Intrinsically, it is generally the most robust combination overall. Moreover, it is the most robust in the forward problems in youth and childhood obesity (44), type I diabetes mellitus (13), and COPD (11). CFP3 is frequently the most statistically significant combination, based on statistical significance alone.

This has been demonstrated for both forward (11, 13, 39) and inverse (10) problems. The critical factor regarding the significance of the results is data length. We would advise a data length of at least 900 RR intervals. This is pertinent here as in some datasets the number of RR intervals was exactly 256 hence about 5 min of time-series when undergoing the task. Despite a potential sparse data hazard the correct levels of significance were achieved (*p* < 0.01; <1%). There is a difference between number of RR intervals in both groups for rest and task. Task time was reduced compared to rest time owing to muscle fatigue that could be caused in the DMD group, as this is a characteristic of the disease.

Future algorithmic manipulations could involve varying the DPSS (45) settings or adjusting Thomson's non-linear combination methods (31) to maximize discrimination. Lastly, weightings of these CGT could be modified as here they are set at unity. This is particularly appropriate for the CFP1 and CFP3 combinations.

Given that DMD subjects benefit from assistive technology; social inclusion, participation, and development—the necessary physiological responses from the technology must be assessed with the possible risk of cardiac, respiratory, dynamical disease (46, 47) states, and general physiological decline. Yet, we understand that future studies should evaluate the HRV recovery from computer tasks so as to state if ANS recovers (or not) from activity, and also to measure if there are positive posterior effects on HRV, as occurs with chronic response of blood pressure after exercises. So, our results support the detection of autonomic responses in DMD during a computer task, to permit the planning of an advantageous clinical management of this group of patients.

Some limitations from this study should be highlighted:

(1) The use of pharmacotherapies could influence our results; beta-blockers and angiotensin-converting enzyme (ACE) inhibitors could interfere in autonomic functions, but these medications are often prescribed and their cessation is not medically or ethically possible, so patients included in the study continued to take these medications. Similarly, we performed a regression analysis so as to study if these medications interfered with our outcomes and did not find any significant results. Besides, we encourage additional studies to evaluate the early effects of autonomic dysfunction in DMD children during the computer tasks; especially those without medication.
(2) To obtain HRV data at different stages of the disease and to better characterize the population; various degrees of pathology (Vignos scale 1–9) were included;
(3) We did not enforce any breathing rhythm and/or emotional analysis; those data in DMD and control group provided useful information and could be important for future studies.

## Conclusion

DMD subjects presented a lower non-linear HRV compared to TD subjects at rest and during a computer task. Both groups exhibited a similar pattern of reduced non-linear HRV during computer tasks with sustained attention demand when measured by the CGT. It demonstrates that although there is an impaired HRV in subjects with DMD, there remains an adaptation of the ANS throughout the computer task. The identification of autonomic impairment is critical when considering the patients' ANS responses, taking into consideration that the computer tasks in the DMD community may elevate their level of social inclusion, participation, and independence. Hence, assistive technologies should be organized in combination with continuous physiological monitoring to lessen serious and possibly hazardous effects.

## Data Availability Statement

The raw data supporting the conclusions of this article will be made available by the authors, without undue reservation. The Matlab code is unavailable due to commercial reasons.

## Ethics Statement

The studies involving human participants were reviewed and approved by Ethics Committee of the University of São Paulo, reference number 236/13. Written informed consent to participate in this study was provided by the participants' legal guardian/next of kin.

## Author Contributions

MA and TS collected data and performed conduction of experiments. MA, CM, TS, VV, CF-F, LV, CF, and DG drafted the manuscript. MA followed the journal guidelines. DG and TS performed statistical analysis. CM supervised the study. MA and CM gave final approval for the version submitted for publication. DG and AS extensively reviewed the manuscript, English Grammar, and spelling. All authors reviewed and approved the manuscript.

## Funding

CM and TS received financial support from FAPESP (Fundação de Amparo à Pesquisa do Estado de São Paulo), process numbers 2012/16970-6 and 2017/24991-7 (CM) and 2016/08358-0 (TS).

## Conflict of Interest

The authors declare that the research was conducted in the absence of any commercial or financial relationships that could be construed as a potential conflict of interest.

## Publisher's Note

All claims expressed in this article are solely those of the authors and do not necessarily represent those of their affiliated organizations, or those of the publisher, the editors and the reviewers. Any product that may be evaluated in this article, or claim that may be made by its manufacturer, is not guaranteed or endorsed by the publisher.
